# Gender Differences in Nutritional Quality and Consumption of Lunches Brought from Home to School

**DOI:** 10.3390/ijerph182413168

**Published:** 2021-12-14

**Authors:** Siwan Song, Ariun Ishdorj, Jayna M. Dave

**Affiliations:** 1 Department of Agricultural Economics, Texas A&M University, 2124 TAMU, College Station, TX 77843, USA; ptswsong@tamu.edu; 2 USDA/ARS Children’s Nutrition Research Center, Baylor College of Medicine, Houston, TX 77030, USA; jmdave@bcm.edu

**Keywords:** lunches brought from home, consumption, NSLP, nutrient intake, nutritional quality, elementary and intermediate school, gender differences

## Abstract

Gender difference in the lunches brought from home to school and the amount consumed by elementary and intermediate school students were examined using data collected from 12 schools in Texas. The amount and nutritional quality of food items brought and consumed was evaluated, by comparing the results to the 2012 school meal standards, and Dietary Reference Intakes (DRIs). Almost all lunches brought from home contained grain and meat/meat alternatives, and the amount brought and consumed exceeded the NSLP standards. The majority of students did not bring fruits, vegetables, and whole grain foods, but those who brought consumed most of what they brought. Among elementary school students, only 9% of boys and 14% of girls brought vegetables and the amount brought and consumed did not meet the standards. Although carbohydrate and protein consumption were adequate for boys and girls, the intakes of micronutrients and fiber did not meet the requirements across both genders at both school levels. Overall, lunches brought from home were not well balanced and did not meet NSLP standards and DRIs. It is imperative to identify strategies to improve the nutritional quality of lunches brought from home considering gender difference in food choice and educating parents and children on what is a healthy well-balanced lunch.

## 1. Introduction

Children’s eating habits are developed early in life and continue to change across their entire childhood with parents and home environment playing an important role [1,2,3]. As children get older, they spend more time away from parents and home [3,4] and spend a significant portion of their weekday at school and eat at least one meal at school per day. Since most children in the U.S. do not meet the daily recommendation for fruits and vegetables and some key nutrients [5,6], school environments and meal programs can play an important and unique role in positively influencing school-age children’s eating habits [7].

On a typical school day in the U.S., students can either participate in the National School Lunch Program (NSLP) and receive free, reduced price or low-cost full price meals or bring packed lunches from home [8]. The NSLP is the second largest federally funded food and nutrition assistance programs in the US operating in public and nonprofit private schools [8]. In the fiscal year 2019, school cafeterias in the U.S. served nearly 5 billion lunches to over 29 million school-age children [8]. To ensure that meals offered will be of high nutritional quality and further to expect the possibility of reducing risk of chronic diseases and obesity, the new nutrition standards and meal requirements were implemented in 2012 [9,10,11]. However, approximately 40% of school children bring lunches from home [8]. These lunches are not subject to federal standards and not consistently monitored as school lunches are.

The few studies that examined the quality of lunches brought from home have found that these lunches are of lower nutritional quality when compared to NSLP standards [12,13,14] or lunches served at school [15,16,17,18]. Additionally, only a few studies have assessed food consumption in addition to what was brought from home [12,17,18]. This is important as it is possible that more food and beverages were packed for students than were consumed. In addition, part of what was brought could have been discarded, shared, or returned home at the end of the day [13].

Previously, Caruso and Cullen [12] studied the quality and cost of lunches brought from home using observational data. They compared lunches brought from home with NSLP nutrition guidelines for elementary and intermediate school students and found that lunches brought from home contained more sodium, fewer servings of fruits, vegetables, whole grains, and fluid milk. Additionally, they reported that desserts, and snack chips, were the food categories with highest average consumption and vegetables had the lowest average consumption.

Existing studies have found differences in food choices and intake by gender; those studies have reported that boys and men eat fewer fruits and vegetables, high-fiber foods, and low-fat foods [19,20,21]. In addition, a major gender difference has been discovered in the practice of restricting food intake as a response to concern about body shape and size [22]. However, most previous studies have not assessed the gender differences in nutritional quality of children’s lunches brought from home and consumed.

The objective of this study was to assess the gender differences in the food items, amount, and nutritional quality of lunches brought from home and the amount consumed by students by comparing the results to the 2012 NSLP standards and DRIs.

## 2. Materials and Methods

### 2.1. Design, Data Collection, and Setting

The data analysis was conducted using the data collected from 12 schools (8 elementary and 4 intermediate schools) in one school district in Houston, Texas, between 6 October 2011, and 5 December 2011. The school district had 39,037 students (26.0% Hispanic, 8.3% African American, 13.4% Asian, 52.3% white) in 26 elementary (kindergarten–grade 5) and 10 intermediate schools (grade 6–8) [12]. Of the 12 schools from which the data were collected, there were 4 low-income and 4 middle-income elementary schools and 2 low-income and 2 middle-income intermediate schools. No identifying information for students was collected. This study was approved by the Baylor College of Medicine Institutional Review Board for Human Subject Research.

Seven trained research staff attended a 3-h cafeteria training session to review procedures and learn how to use the meal observation form. The form listed common lunch food items (sandwiches, fruit, vegetables, drinks, desserts, and snack foods) in the first column, and contained additional columns to check off the food item the student brought, write additional information, add items not on the form, amount of food items that were consumed, and food items that were obtained from other sources. Each observer recorded 2 to 4 practice observations and the research coordinator also collected consumption. One trained observer conducted quality control checks once per month.

Observers were assigned to specific schools that they were to visit unannounced, once per week. They observed unobtrusively from a distance. Foods brought from home and amount consumed were recorded on the observation sheets during lunch period; student’s grade level and gender was also noted. In addition, food obtained from other sources, given away, or spilled was also recorded by observers. Data were entered into the Nutrition Data System for Research (NDSR) diet analysis software (version 2011; Nutrition Coordinating Center, University of Minnesota, Minneapolis, MN, USA). Additional details on data collection procedures can be found in Caruso and Cullen [12].

### 2.2. Statistical Analysis

The descriptive statistics were used to describe the food and nutritional content of lunches brought from home to school and amount consumed using the data from all the students in the sample and for subsample of students who brought the specific food item. Mean and standard deviations for amount of food and nutrients were calculated. The quality of lunches brought from home was evaluated by comparing the amount and serving of foods and beverages brought from home with NSLP standards. The food groups (fruits, vegetables, whole grains, meat/meat alternative milk) and nutrient content (sodium, saturated fat, energy, vitamins, elements, and macronutrients) were compared with NSLP and 33% of the age and gender appropriate DRIs [23]. For the mean intakes and comparison with NSLP standards, student grade levels were used, since the NSLP standards require schools to use age-grade groups [9]. For comparison of mean intakes with DRI, age groups were used, since DRI value vary by age and gender. For sodium limits, school year 2014–2015 requirements were used since the study data were collected in 2011. The Shapiro–Wilk method was used to test the normality of the data, and for normally distributed data, the t-tests were conducted to determine whether there were statistically significant differences in mean amount of food and nutrient contents of lunches brought from home and consumed by gender. Differences were considered statistically significant at *p*-value < 0.05. All statistical analyses were performed using Python (v 3.9.0).

## 3. Results

A total of 337 students who brought lunches from home were observed. Three observations were dropped due to missing gender information. Therefore, a total of 334 lunches brought from home and consumed were used in the analysis, of which 239 (72%) were from elementary schools and 95 (28%) were from intermediate schools. About 49% of all students in the sample were male.

### 3.1. Results for All Students Who Brought Lunch from Home as Related to NSLP Requirements

As outlined in Table 1 and Table 2, for all the students who brought lunches from home, the amounts of total grains and meat/meat alternatives brought exceeded the NSLP requirements. However, the amount brought did not meet the standards for vegetables and milk. For example, the amount of vegetables brought from home to school by elementary school boys was only 11 percent and by intermediate school boys, was 7 percent of the NSLP requirement. Both elementary and intermediate school students brought amounts of fruit that were close to NSLP requirements. Significantly more amount of snack chips was brought from home by elementary school boys compared to girls (*p* = 0.040). Intermediate school girls brought significantly more vegetables compared to boys (*p* = 0.047) and the amount exceeded four times the amount brought by boys. Conversely, intermediate school boys brought significantly more whole grains (*p* = 0.025) and meat/meat alternatives (*p* = 0.002) from home compared to girls. The lunches brought from home met the NSLP requirements for % saturated fat and sodium for all grade level. Moreover, the lunches brought from home met the NSLP standards for energy for elementary school girls and intermediate school boys and were slightly below the standards for the intermediate school girls (Table 3). Intermediate school boys brought foods that were significantly higher in the amounts of calories (*p* = 0.044) and sodium (*p* = 0.013) compared to girls.

For both elementary and intermediate school students, the amounts of fruit, vegetables, and milk consumed did not meet the NSLP requirements. Additionally, whole grain intake was not met among elementary school students as well as intermediate school girls. Almost all sweetened beverages, snack chips, and desserts brought from home were consumed. The amount of snack chips and desserts consumed by intermediate school students exceeded the amount brought from home, since some students purchased a la carte foods. Elementary school girls consumed significantly more amounts of fruit brought from home compared to boys (*p* = 0.047). However, elementary school boys consumed significantly more amounts of total grains (*p* = 0.046) and snack chips (*p* = 0.023) compared to girls. Intermediate school boys consumed significantly higher amounts of total grains (*p* = 0.009), whole grains (*p* = 0.010), and meat/meat alternatives (*p* = 0.000) compared to girls. Significant gender differences in energy intake was observed for elementary (*p* = 0.044) and intermediate (*p* = 0.001) school students, and in sodium intakes (*p* = 0.001) for intermediate school students only. On average, elementary and intermediate school girls’ energy intakes were below the NSLP minimum requirements for energy.

### 3.2. Results for Students Who Brought Specific Food Groups from Home as Related to NSLP Requirements

As reported in Table 1 and Table 2, milk was the least common lunch food item brought from home to school (elementary—10% boys and 6% girls; intermediate—0% boys and 4% girls) followed by vegetables (elementary—9% boys and 14% girls; intermediate—16% boys and 21% girls), and whole grains (elementary—34% boys and 28% girls; intermediate —56% boys and 44% girls). Over half of lunches brought from home by elementary school students contained fruits, sweetened beverages, snack chips, and desserts and almost all lunches contained grains and meat/meat alternatives. About forty percent of lunches containing grains were whole grains (Elementary—34% boys and 28% girls, intermediate –57% boys and 45% girls). Among students who brought foods from the specific food groups, the mean amount of food brought and consumed exceeded the NSLP standards, except for vegetables, and those who brought, consumed most of what they brought. For example, among the intermediate school boys who brought vegetables to school, the amount brought was 40 percent of the NSLP requirements; however, they consumed all the vegetables they brought.

Although the gender difference for fruit brought from home was not significant, the elementary school girls consumed significantly higher amounts of fruit than boys did (*p* = 0.042). Conversely, elementary school boys consumed significantly more total grains than girls did (*p* = 0.047). Intermediate school girls brought significantly more vegetables from home than did boys (*p* = 0.036), although there was no significant difference in the consumption. Intermediate school boys consumed significantly high amounts of total grains than girls (*p* = 0.005). Moreover, compared to girls, intermediate school boys brought and consumed significantly more whole grain foods, meat/meat alternatives, and snack chips (*p* < 0.05).

### 3.3. Results for All Students Who Brought Lunches from Home as Related to DRI

Nutrient contents of lunches brought from home were compared with DRI. As shown in Table 4, all the lunches brought from home were below 33% DRI for vitamin D and potassium; over 90% of lunches brought by 9 to 13-year-olds were below 33% DRI in calcium. Over half of lunches brought by 5 to 8-year-olds met the 33% DRI for vitamin C, vitamin B12, folate, and iron.

On average, the mean intake of vitamins C was sufficient when compared with recommended DRIs However, intakes of vitamins D and A, calcium and potassium were lower than 33% of age-appropriate DRI among all students. Mean amount of iron intake was met among 9–13 years old students only but not for 5 to 8-year-olds. Carbohydrate and protein intakes from lunches brought from home exceeded the age-appropriate DRIs (Table 5). Intake of fiber was less than 33% of the DRIs for students of all ages.

There was no significant difference in mean amount of nutrients between boys and girls among 5–8 years old students. Mean amount of vitamin B12, minerals and macronutrients consumed by students aged 9–13 was significantly different between boys and girls (*p* < 0.05).

## 4. Discussion

To our knowledge, the current study is one of the few to examine gender differences in the food content, amount, and nutritional quality of lunches brought from home and amount consumed. In keeping with the results of previous studies in the U.S. and United Kingdom [12,13,14,15,16,17], it was found that lunches brought from home had lower nutritional quality when compared to NSLP standards and age-appropriate DRIs. In addition, there were significant differences in mean amounts of whole grain, meat, and snack chips consumed between genders. The lunches brought by most students contained mainly grains and meat/meat alternatives, and the mean amounts of grains and meat/meat alternatives brought and consumed tend to be at least twice the minimum amount required by NSLP guidelines. Since grains and meats are main sources of carbohydrates and protein, respectively, our findings are in line with the findings from the existing literature where it was consistently shown that, on average, all students in the various age groups exceeded the DRIs in carbohydrate and protein consumption [12,15,17,24]. However, among the students who brought any grains, whole grains were brought by 40% of students. This finding reveals that the intake of whole grains remains low regardless of gender. Therefore, to encourage healthy sources of carbohydrates, more attention to promoting whole grain food consumption is warranted.

Consistent with previous studies [12,15,16,25], the current study also found that mean amounts of fruit and vegetables in lunches brought from home to school were not sufficient and did not meet NSLP guidelines. Among those students who brought fruits and vegetables, the amount of fruit consumed met NSLP standards, but only 40% of students brought fruits. The amount of vegetables consumed did not meet the standards. This explains the low intake of dietary fiber found in this study, since fruits, vegetables, and whole grains are the main sources of dietary fiber. Of those who brought vegetables to school, the intermediate school girls brought significantly more vegetables than boys, and the amount brought exceeded the NSLP standards. However, boys consumed all the vegetables they brought whereas girls consumed only 63% of what they brought. The findings of the current study reveal that preferences in foods brought from home to school and consumed not only differed by gender, but gender differences varied by school level. Therefore, accounting for gender and age differences in food preferences when examining lunches brought from home as well as the consumption of lunches provided by NSLP is important to further understand the factors affecting children’s food choices and preferences. Since NSLP lunches were found to be more nutritious than lunches brought from home [15,25], further assessment to explore the reasons for bringing lunches from home rather than opting for school lunches is warranted.

The levels of vitamin C were high in lunches brought from home, possibly due to fortified sugar-sweetened beverages, as repeatedly shown in previous studies [15,26]. Alternatively, the lunches brought from home could include vitamin C rich foods such as citrus fruits, broccoli, peppers, leafy greens, etc. Levels of vitamins D and A were inadequate in lunches brought from home. In addition, consumed amount of nutrients was different between boys and girls since their food choice and preferences were different. Boys consumed more vitamin D and calcium than girls, although the mean amount consumed by boys is still low compared to DRI. It is important to address unbalanced nutritional intake by promoting to pack healthier options. Lunches brought from home also lacked fundamental elements, including calcium, iron, and potassium. The lack of calcium and vitamin A reflects the fact that milk was brought by few students (6.3%) and elementary school students who brought milk and consumed met the NSLP standards. These findings suggest a need to focus on promotion of water and low-fat milk rather than bringing and consuming sugar-sweetened beverages. However, as food safety standards dictate, milk should be stored at 40 °F or below until consumed, hence maintaining the beverage at a safe temperature could be a challenge when milk is brought from home.

Unlike the results of previous studies [17,25], lunches brought from home did not lead to increased caloric intake. Mean amount of energy consumed by intermediate school girls was lower than the recommended levels. This is consistent with studies on gender difference in food choice or preferences, that girls have greater concern about weight and become pickier as they get older [20,21]. On average, lunches brought from home did not exceed sodium level of NSLP guidelines unlike in previous studies [15,27,28]. However, although the mean amounts of sodium in lunches brought from home met the school year 2014–2015 requirements, it did not meet the sodium intake level of NSLP guidelines, if compared with the final target of sodium intake level set for school year 2022–2023. This indicates that parents should be informed of the requirements and how they can pack lunches containing foods lower in sodium. Minaya et al. [29] suggested that nutrition educators should host parent meetings that showcase new recipes incorporating lower sodium options through use of more herbs and spices, and parents should look for snacks or vegetables with low sodium or no salt added.

The most notable strength of this study was the use of actual consumption data on lunches brought from home to school for both elementary and intermediate school students and assessment of the nutritional quality of these lunches by gender. We were thus able to document students’ actual consumption and report results for all the students in our sample as well as for those who brought the food item. The findings can serve as a basis for encouraging parents to focus on variety of foods when packing lunch and help educators in designing appropriate nutrition education and health promotion classes for parents, and students. This will help ensure that students eat healthy lunches since previous studies on interventions observed some improvements in food and nutrient content of meals brought from home to school for lunch [30,31]. In addition, Farris et al. [15] urged that parents involve their children in meal planning and give them autonomy in choosing vegetables, like between baby carrots and sliced cucumbers. A limitation of this study is that these findings cannot be generalized to a broader sample of students, as data from one-day packed lunch was restricted to southeast Texas area. It is possible that students consume enough nutrients at home and during the other days of the week. Nonetheless, parents need to be aware of the importance of packing healthy foods for their children. Future studies could improve upon this by using multiple-day periods of observations to capture more reliable estimates in line with the new NSLP standards, which also provide guidance over a 5-day period. Additionally, future research should consider variations in food environment characteristics of different schools to compare different dietary behaviors between students who eat school lunches and students who eat lunches brought from home.

## 5. Conclusions

Understanding the reasons the students choose to bring lunches from home to school, who is packing their lunches, and why they are packing certain foods over others will help find effective ways in positively influencing parents and students’ food choices and consumption. In the present study, we found that on an average, lunches brought from home to school tend not to be nutritionally balanced when compared to NSLP guidelines and DRIs. The majority of students did not bring milk, fruits, vegetables, and whole grain foods to school, but those who brought, consumed most of what they brought. Therefore, parents and students should be encouraged to pack more of these foods for lunch. In addition, this study found that nutritional quality of lunches brought from home and consumed differed by gender and these differences varied by school level. Developing nutrition education materials and interventions that are age and gender tailored can be effective in improving nutritional quality of lunches brought from home and consumed. Furthermore, increasing enrollment in the NSLP could help ensure that more students receive nutritionally balanced lunches.

## Figures and Tables

**Table 1:** Mean amounts of food brought from home and consumed by elementary school students compared with NSLP guidelines, by food groups and gender.

		Elementary School					
		All Students			Students Brought Food Item		
Food Groups		Boys	Girls	*p*-Value	Boys	Girls	*p*-Value
Total Fruit, cups	NSLP Guideline	0.5			0.5		
	n (%)	121	118		50 (41.3)	55 (46.6)	
	Brought ^a^ (% ^b^)	0.44 ± 0.57 (88.0)	0.57 ± 0.70 (114.0)	0.121	1.06 ± 0.37 (212.0)	1.37 ± 0.77 (274.0)	0.081
	Consumed ^a^ (% ^b^)	0.27 ± 0.44 (54.0)	0.39 ± 0.56 (78.0)	0.047	0.66 ± 0.46 (132.0)	0.82 ± 0.66 (164.0)	0.042
Total Vegetables, cups	NSLP Guideline	0.75			0.75		
	n (%)	121	118		11 (9.1)	16 (13.6)	
	Brought ^a^ (% ^b^)	0.08 ± 0.31 (10.7)	0.10 ± 0.30 (13.3)	0.615	0.86 ± 0.65 (114.7)	0.73 ± 0.48 (97.3)	0.535
	Consumed ^a^ (% ^b^)	0.06 ± 0.27 (8.0)	0.07 ± 0.25 (9.3)	0.779	0.66 ± 0.65 (88.0)	0.52 ± 0.51 (69.3)	0.509
Total Grain, oz eq.	NSLP Guideline	1≤			1≤		
	n (%)	121	118		120 (99.2)	118 (100.0)	
	Brought ^a^ (% ^b^)	2.90 ± 1.30 (290.0)	2.69 ± 1.18 (269.0)	0.206	2.92 ± 1.27 (292.0)	2.69 ± 1.18 (269.0)	0.154
	Consumed ^a^ (% ^b^)	2.44 ± 1.29 (244.0)	2.13 ± 1.26 (213.0)	0.046	2.45 ± 1.27 (245.0)	2.12 ± 1.26 (212.0)	0.047
Whole Grain, oz eq.	NSLP Guideline	0.5			0.5		
	n (%)	121	118		41 (33.9)	33 (28.0)	
	Brought ^a^ (% ^b^)	0.46 ± 0.38 (92.0)	0.42 ± 0.77 (84.0)	0.712	1.35 ± 0.63 (270.0)	1.50 ± 0.71 (300.0)	0.318
	Consumed ^a^ (% ^b^)	0.38 ± 0.69 (76.0)	0.38 ± 0.70 (76.0)	0.987	1.14 ± 0.73 (228.0)	1.36 ± 0.63 (272.0)	0.16
Meat/meat alternatives, oz eq.	NSLP Guideline	1≤			1≤		
	n (%)	121	118		119 (98.3)	117 (99.2)	
	Brought ^a^ (% ^b^)	1.87 ± 0.77 (187.0)	1.87 ± 0.88 (187.0)	0.951	1.91 ± 0.73 (191.0)	1.88 ± 0.87 (188.0)	0.833
	Consumed ^a^ (% ^b^)	1.52 ± 0.88 (152.0)	1.46 ± 0.92 (146.0)	0.618	1.54 ± 0.86 (154.0)	1.47 ± 0.92 (147.0)	0.539
Total Milk, cups	NSLP Guideline	1			1		
	n (%)	121	118		12 (9.9)	7 (5.9)	
	Brought^a^ (% ^b^)	0.10 ± 0.31 (10.0)	0.07 ± 0.28 (7.0)	0.466	0.97 ± 0.33 (97.0)	1.14 ± 0.38 (114.0)	0.296
	Consumed ^a^ (% ^b^)	0.11 ± 0.33 (11.0)	0.10 ± 0.35 (10.0)	0.848	0.90 ± 0.51 (90.0)	1.29 ± 0.49 (129.0)	0.124
Sweetened Beverage, oz	NSLP Guideline	NA			NA		
	n (%)	121	118		60 (49.6)	74 (62.7)	
	Brought ^a^	4.05 ± 4.97	4.82 ± 4.58	0.220	8.18 ± 4.00	7.68 ± 3.36	0.435
	Consumed ^a^	3.57 ± 4.12	4.17 ± 3.91	0.245	7.19 ± 2.82	6.65 ± 2.79	0.269
Snack chips, servings	NSLP Guideline	NA			NA		
	n (%)	121	118		71 (58.7)	54 (45.8)	
	Brought ^a^	0.67 ± 0.67	0.50 ± 0.63	0.040	1.15 ± 0.46	1.09 ± 0.47	0.511
	Consumed ^a^	0.62 ± 0.61	0.45 ± 0.57	0.023	1.04 ± 0.46	0.97 ± 0.46	0.422
Desserts, servings	NSLP Guideline	NA			NA		
	n (%)	121	118		70 (57.9)	70 (59.3)	
	Brought ^a^	0.70 ± 0.84	0.64 ± 0.72	0.563	1.20 ± 0.79	1.08 ± 0.64	0.288
	Consumed ^a^	0.65 ± 0.76	0.59 ± 0.70	0.506	1.10 ± 0.70	0.97 ± 0.68	0.263

^a^ Mean amount ± Standard deviation (SD). ^b^ Percentage of NSLP guideline requirements and calculations based on mean amount.

**Table 2:** Mean amounts of food brought from home and consumed by intermediate school students compared with NSLP guidelines, by food groups and gender.

			Intermediate School				
		All Students			Students Brought Food Item		
Food Groups		Boys	Girls		Boys	Girls	
Total Fruit, cups	NSLP Guideline	0.5			0.5		
	n (%)	43	52		20 (46.5)	20 (38.5)	
	Brought ^a^ (% ^b^)	0.64 ± 0.86 (128.0)	0.40 ± 0.55 (80.0)	0.111	1.37 ± 0.77 (274.0)	1.04 ± 0.35 (208.0)	0.092
	Consumed ^a^ (% ^b^)	0.38 ± 0.61 (76.0)	0.32 ± 0.50 (64.0)	0.555	0.82 ± 0.66 (164.0)	0.82 ± 0.49 (164.0)	0.990
Total Vegetables, cups	NSLP Guideline	0.75			0.75		
	n (%)	43	52		7 (16.3)	11 (21.2)	
	Brought ^a^ (% ^b^)	0.05 ± 0.12 (7.0)	0.21 ± 0.55 (28.0)	0.047	0.30 ± 0.14 (40.0)	1.01 ± 0.81 (134.7)	0.036
	Consumed ^a^ (% ^b^)	0.09 ± 0.24 (12.0)	0.14 ± 0.48 (18.7)	0.562	0.30 ± 0.14 (40.0)	0.64 ± 0.90 (85.3)	0.336
Total Grain, oz eq.	NSLP Guideline	1≤			1≤		
	n (%)	43	52		42 (100.0)	51 (98.1)	
	Brought ^a^ (% ^b^)	2.90 ± 0.95 (290.0)	2.56 ± 1.28 (256.0)	0.158	2.96 ± 0.85 (296.0)	2.61 ± 1.24 (261.0)	0.119
	Consumed ^a^ (% ^b^)	2.97 ± 1.09 (297.0)	2.32 ± 1.26 (232.0)	0.009	3.04 ± 1.00 (304.0)	2.37 ± 1.23 (237.0)	0.005
Whole Grain, oz eq.	NSLP Guideline	0.5			0.5		
	n (%)	43	52		24 (55.8)	23 (44.2)	
	Brought ^a^ (% ^b^)	0.82 ± 0.97 (164.0)	0.46 ± 0.58 (92.0)	0.025	1.47 ± 0.84 (294.0)	1.03 ± 0.40 (206.0)	0.028
	Consumed ^a^ (% ^b^)	0.82 ± 0.91 (164.0)	0.42 ± 0.55 (84.0)	0.010	1.39 ± 0.81 (278.0)	0.95 ± 0.43 (190.0)	0.022
Meat/meat alternatives, oz eq.	NSLP Guideline	1≤			1≤		
	n (%)	43	52		41 (95.3)	44 (84.6)	
	Brought ^a^ (% ^b^)	1.96 ± 1.02 (196.0)	1.36 ± 0.83 (136.0)	0.002	2.05 ± 0.95 (205.0)	1.61 ± 0.64 (161.0)	0.012
	Consumed ^a^ (% ^b^)	2.13 ± 1.40 (213.0)	1.14 ± 0.76 (114.0)	0.000	2.24 ± 1.34 (224.0)	1.35 ± 0.64 (135.0)	0.000
Total Milk, cups	NSLP Guideline	1			1		
	n (%)	43	52		0 (0.0)	2 (3.8)	
	Brought ^a^ (% ^b^)	0.00 ± 0.00 (0.0)	0.27 ± 0.16 (27.0)	0.261	0.00 ± 0.00 (0.0)	0.70 ± 0.55 (70.0)	N/A
	Consumed ^a^ (% ^b^)	0.02 ± 0.15 (2.0)	0.03 ± 0.15 (3.0)	0.967	0.00 ± 0.00 (0.0)	0.64 ± 0.57 (64.0)	N/A
Sweetened Beverage, oz	NSLP Guideline	NA			NA		
	n (%)	43	52		19 (44.2)	19 (36.5)	
	Brought ^a^	3.75 ± 4.96	3.76 ± 5.73	0.988	8.48 ± 3.88	10.30 ± 4.70	0.201
	Consumed ^a^	3.74 ± 4.96	3.00 ± 4.87	0.463	8.48 ± 3.88	8.22 ± 4.69	0.849
Snack chips, servings	NSLP Guideline	NA			NA		
	n (%)	43	52		28 (65.1)	36 (69.2)	
	Brought ^a^	0.81 ± 0.80	0.68 ± 0.51	0.350	1.24 ± 0.67	0.98 ± 0.26	0.038
	Consumed ^a^	0.85 ± 0.78	0.66 ± 0.50	0.167	1.24 ± 0.67	0.96 ± 0.26	0.024
Desserts, servings	NSLP Guideline	NA			NA		
	n (%)	43	52		24 (55.8)	23 (44.2)	
	Brought ^a^	0.50 ± 0.64	0.49 ± 0.70	0.897	0.90 ± 0.60	1.10 ± 0.65	0.288
	Consumed ^a^	0.55 ± 0.61	0.60 ± 0.77	0.754	0.91 ± 0.53	1.04 ± 0.55	0.395

^a^ Mean amount ± Standard deviation (SD). ^b^ Percentage of NSLP guideline requirements and calculations based on mean amount.

**Table 3:** Mean amounts of nutrients brought from home and consumed compared with NSLP standards.

School Type	Nutrient Content	NSLP Guidelines	Amount Brought Mean ± SD			Amount Consumed Mean ± SD		
			Boy	Girl	*p*-Value	Boy	Girl	*p*-Value
Elementary	Energy, kcal	550–650	677.9 ± 189.9	644.5 ± 184.3	0.168	584.5 ± 186.7	533.6 ± 203.2	0.044
	Saturated fat, %	<10	9.7 ± 3.9	10.1 ± 3.7	0.311	9.41 ± 4.4	10.1 ± 4.1	0.426
	Sodium, mg	<= 1230 ^a^	1131.1 ± 483.3	1090.0 ± 441.0	0.491	950.4 ± 496.6	867.7 ± 450.1	0.179
Intermediate	Energy, kcal	600–700	659.8 ± 201.1	579.32 ± 216.8	0.044	689.74 ± 240.1	537.4 ± 208.5	0.001
	Saturated fat, %	<10	9.3 ± 3.6	8.77 ± 3.8	0.711	9.6 ± 3.4	8.8 ± 4.3	0.426
	Sodium, mg	<= 1360 ^a^	1131.8 ± 456.1	897.28 ± 446.1	0.013	1243.9 ± 664.3	797.8 ± 435.8	0.000

^a^ For sodium limits, SY 2014–2015 requirements were used.

**Table 4:** Comparison of mean amounts of vitamins and minerals from lunches brought from home with age-appropriate DRIs.

Nutrient	33% DRI		Number of Lunches Brought from Home < 33% DRI		Nutrient Consumed Mean ± SD		
	Boy	Girl	Boy (%)	Girl (%)	Boy	Girl	*p*-Value
Age 5–8 (Boy: *n* = 81, Girl: *n* = 79)							
Vitamin C (mg/d)	8.3	8.3	38 (48)	35 (44)	15.8 ± 22.3	18.2 ± 29.9	0.469
Vitamin D (μg/d)	5.0	5.0	81 (100)	79 (100)	0.7 ± 0.9	0.6 ± 1.0	0.829
Vitamin B12 (μg/d)	0.4	0.4	47 (58)	42 (53)	0.5 ± 0.4	0.5 ± 0.5	0.747
Vitamin A (μg/d)	132.0	132.0	57 (70)	54 (68)	99.5 ± 110.3	103.1 ± 103.7	0.846
Folate (μg/d)	66.0	66.0	26 (32)	26 (33)	72.9 ± 37.1	67.2 ± 35.9	0.308
Calcium (mg/d)	330.0	330.0	53 (65)	58 (73)	229.8 ± 179.5	217.0 ± 157.4	0.611
Iron (mg/d)	3.3	3.3	39 (48)	38 (48)	3.0 ± 1.7	2.8 ± 1.4	0.322
Potassium (mg/d) *	1300	1300	81 (100)	78 (99)	409.0 ± 231.5	412.8 ± 220.9	0.895
Age 9–13 (Boy: *n* = 83, Girls: *n* = 91)							
Vitamin C (mg/d)	14.9	14.9	54 (65)	59 (66)	18.9 ± 31.9	16.2 ± 24.4	0.536
Vitamin D (μg/d)	5.0	5.0	83 (100)	91 (100)	0.5 ± 0.8	0.3 ± 0.6	0.144
Vitamin B12 (μg/d)	0.6	0.6	57 (69)	75 (83)	0.5 ± 0.6	0.3 ± 0.4	0.004
Vitamin A (μg/d)	198.0	198.0	75 (90)	80 (88)	85.5 ± 102.8	94.5 ± 140.4	0.631
Folate (μg/d)	99.0	99.0	37 (46)	46 (51)	104.9 ± 41.9	86.1 ± 43.0	0.004
Calcium (mg/d)	429.0	429.0	76 (92)	84 (92)	235.4 ± 170.1	189.2 ± 129.9	0.044
Iron (mg/d)	2.6	2.6	14 (18)	19 (21)	4.0 ± 1.6	3.2 ± 1.4	0.000
Potassium (mg/d) *	1500	1500	83 (100)	91 (100)	495.0 ± 246.1	415.2 ± 242.7	0.032

* Adequate Intake.

**Table 5:** Comparison of mean amounts of macronutrients from lunches brought from home with age-appropriate DRIs.

Nutrient	33% DRI		Number of Lunches Brought from Home < 33% DRI		Nutrient Consumed Mean ± SD		
	Boy	Girl	Boy (%)	Girl (%)	Boy	Girl	*p*-Value
Age 5–8 (Boy: *n* = 81, Girl: *n* = 79)							
Carbohydrate (g/d)	42.9	42.9	3 (4)	2 (3)	78.0 ± 27.0	73.8 ± 28.5	0.416
Dietary Fiber (g/d)	8.3	8.3	77 (94)	72 (90)	4.3 ± 2.4	5.4 ± 2.8	0.790
Protein (g/d)	6.3	6.3	1 (1)	1 (1)	15.8 ± 7.4	14.7 ± 7.2	0.359
Age 9–13 (Boy: *n* = 83, Girls: *n* = 91)							
Carbohydrate (g/d)	42.9	42.9	2 (3)	8 (9)	95.1 ± 30.2	80.4 ± 33.9	0.003
Dietary Fiber (g/d)	10.2	8.6	80 (96)	85 (93)	5.4± 2.8	4.9 ± 2.6	0.027
Protein (g/d)	11.2	11.2	7 (8)	13 (14)	20.4 ± 10.8	15.8 ± 8.1	0.001

## Data Availability

The data presented in this study are available on request from the corresponding author. The data are not publicly available due to privacy restrictions.
